# One-month comparative efficacy of three topical ectoparasiticides against adult brown dog ticks (*Rhipicephalus sanguineus* sensu lato) on mixed-bred dogs in controlled environment

**DOI:** 10.1007/s00436-015-4356-8

**Published:** 2015-02-07

**Authors:** Marie Varloud, Josephus J. Fourie

**Affiliations:** 1 Ceva Santé Animale S. A, 10 Avenue de la Ballastière, 33500 Libourne, France; 2 ClinVet International (Pty) Ltd, P.O. Box 11186, Universitas, 9321 South Africa

**Keywords:** Acaricidal efficacy, Permethrin, Fipronil, *Canis familiaris*, Treatment, Prevention

## Abstract

This study was designed to compare the therapeutic and residual efficacy for 1 month of three topical ectoparasiticides on mixed-bred dogs against the brown dog tick, *Rhipicephalus sanguineus*. Adult dogs (*n* = 32, 10.8–18.4 kg BW) were allocated to 4 groups (*n* = 8) and infested with 50 adult ticks on days −8, −2, 7, 14, 21, and 28. Within each group, dogs were treated topically on day 0 with a control solution (CS), Vectra® 3D (DPP), Frontline® Plus (FM), or K9 Advantix® (IP). Ticks were enumerated on dogs 24 h after treatment and each subsequent tick infestation by *in situ* thumb count assessment without removal and at 48 h by combing and removal. Acaricidal efficacy was calculated using arithmetic means for all 24 and 48 h tick count assessments. From 42 to 56 % of the total, infested ticks were found on dogs 48 h post-challenge in the CS group. Therapeutic efficacy for all treatments ranged from 45.5 to 64.6 % after 48 h of infestation. Residual efficacy after FM treatment was consistently lower compared to DPP or IP treatments at the 24 h assessments on days 8, 22, 23, and 29. Residual efficacy measured at this last time point was 94.8 % for DPP, 83.1 % for IP, and 46.9 % for FM. This study demonstrates that permethrin-based formulations (DPP and IP) provided a quicker onset of residual protection against brown dog ticks compared to FM. Although DPP and IP are both permethrin-based formulations, DPP exhibited consistently higher residual acaricidal efficacies and was the only treatment that provided >90 % protection for 1 month at 24 h post challenge.

## Introduction

The brown dog tick (*Rhipicephalus sanguineus* sensu lato) is a widespread blood-feeding parasite of mammals, with a strong tropism for dogs. Contrary to other three-host tick species that can infest companion animals, *R. sanguineus* is able to complete each of the feeding stages of his cycle on dogs, including infestations that originate from ticks that are acquired indoors. Adult females feed on their host for several days before dropping and entering in a reproductive process. Each individual female can lay an average of 1,500–4,000 eggs. Unfed larvae, nymphs, and adults can persist in their environment for several months (Dantas-Torres 2010). In humans, *R. sanguineus* is considered as a vector of Crimean-Congo hemorrhagic fever virus (Gergova et al. 2012) and Rickettsiae (Parola et al. 2008). In dogs, it can transmit pathogens responsible for severe diseases: bacteria such as *Ehrlichia canis* (Aguiar et al. 2007), protozoa such as *Hepatozoon canis* (Nordgren and Craig 1984), and helminths such as *Cercopithifilaria bainae* (Ramos et al. 2013). Except *H. canis*, these micro-organisms are mostly transmitted after attachment during the blood-meal step. Rapid removal of already attached ticks and prevention of blood-feeding by this parasite are, therefore, two key points of the prevention strategy against ticks and their associated diseases. In order to achieve this aim, dogs can be treated topically and on a regular basis with different formulations of acaricidal actives like fipronil or permethrin. These actives are usually associated with an insect growth regulator or an insecticidal active in order to enlarge the spectrum of activity to other parasites.

The products currently available to veterinarians and pet owners vary not only regarding actives and their concentrations, but also regarding their overall formulations. Chemistry and quality of ingredients confer to the products a large part of their efficacy properties (Endris et al. 2003). The demonstration of the efficacy of these products is usually performed in field studies or on small populations (*n* ≥ 6) of dogs conducted in highly controlled environments.

## Materials and methods

The animals were not treated by any individual involved in performing the post-treatment assessments and observations. Study groups were coded to blind the assessors.

### Dogs

Thirty-four healthy mongrel dogs (>6 months old), which had not been treated with an acaricide or insecticide for 12 weeks prior to day 0 of the study, started the 10-day acclimation period. All dogs were identified with electronic transponders and were dewormed at the beginning of the study. They were fed commercial dog food once daily with water available *ad libitum*. Within each gender, the dog with the lowest tick count was excluded from the study. There were 32 dogs (1:1 sex ratio), with body weight (BW) ranging from 10.8 to 18.4 kg and with hair length ranging from 12 to 61 mm, enrolled for the study. The dogs were housed individually in an indoor animal unit, controlled for temperature (∼20 ± 4 °C) with 12 h light/12 h darkness photoperiod. No contact between dogs was possible during the study. All dogs were observed for general health status and adverse reactions to treatment once daily, from day −10 to day 30, except on day 0, when specific health observations and local tolerance assessments were made within the 2 h prior to treatment and possible adverse events to treatment were observed 1, 2, and 8 h (±30 min) post-treatment This protocol was approved by an independent animal ethics committee.

### Allocation

The study followed a randomized block design. The 32 dogs included were ranked, within gender, in descending order of individual day −6 tick counts. Within each gender, animals were blocked into blocks of four dogs each. Within each block, dogs were randomly allocated to groups 1 to 4.

### Treatment

Each dog was treated with the allocated treatment on day 0. Dogs in group 1 received the placebo control solution (CS) containing the vehicle of the commercial formulation Vectra® 3D (Ceva Animal Health, Lenexa, KS) with no active ingredients. Dogs in group 2 were treated with the commercial formulation Vectra® 3D (DPP) containing the active ingredients dinotefuran (4.95 %), pyriproxifen (0.44 %), and permethrin (36.08 %). Group 3 was treated with the commercial formulation Frontline® Plus (Merial Ltd., Duluth, GA) containing fipronil (9.8 %) and (S)-methoprene (8.8 %; FM) and group 4 was treated with the commercial formulation K9 Advantix® 55 (Bayer HealthCare, Shawnee mission, Kansas) containing imidacloprid (8.8 %) and permethrin (44.0 %; IP). The products were administered topically, as spot-on, according to the manufacturers’ label directions. CS and DPP were administered at a rate of 3.6 mL per dog applied equally (1.2 mL per site) in three spots at the shoulder blades, the mid-back and the base of tail. FM was administered at a rate of 1.34 mL per dog. IP was administered at a rate of 2.5 mL per dog.

### Tick infestations

A laboratory-bred strain of *R. sanguineus* was used in the experimental infestation. Immature *R. sanguineus* were fed on rabbits. Each dog was infested, under sedation (IV, medetomidine hydrochloride, Domitor®), with 50 ticks on days −8, −2, 7, 14, 21, and 28. The adult ticks were unfed, at least 1 week old (post final ecdysis) and had a balanced sex ratio (1:1).

The infestations were performed by placing ticks along the ventro/lateral aspects of the dog allowing the ticks to disperse within the hair. Tick counts were conducted on dogs *in situ* at 24 ± 1 h and at removal 48 ± 2 h post-infestation or treatment. During the 24-h *in situ* tick count assessments, ticks were found and counted through palpation and visual inspection of the dog’s skin and coat. When a tick was located on the dog, the tick was visually examined to determine if it was live or dead, free, or attached. Ticks were categorized according to categories 1, 2, 4, and 5 (Table 1). During the 48-h count and removal assessments, ticks were found, counted, and removed through palpation and visual inspection of the dog’s skin and coat. All dogs were, however, combed for a minimum of 10 min using a flea comb to make sure all ticks were removed and counted after palpation and visual inspection (Table 1).

**Table 1:** Classification of ticks on their state of attachment and engorgement and whether they are live or not

Category	Condition	Attachment status
1	Live	Unattached
2		Attached; unengorged*
3		Attached, engorged**
4	Dead	Unattached
5		Attached; unengorged*
6		Attached, engorged**

*No filling of the alloscutum obvious

**Obvious or conspicuous filling of the alloscutum

### Statistical analysis

Arithmetic (AM) and geometric means (GM) were calculated. For geometric means, the calculations were based on the means of the tick (count + 1) data. One (1) was subsequently subtracted from the result to obtain a meaningful value for the geometric mean of the study groups.

Percent efficacy for the *in situ* (24 h) count on each treatment group on each day was calculated as:

$$ Efficacy\ 24h\ \left(\%\right)=100\times \frac{\left({\mathrm{MC}}_{24}-{\mathrm{MT}}_{24}\right)}{{\mathrm{MC}}_{24}} $$

Where:

MC_24_: mean (geometric or arithmetic) of live ticks (categories 1 and 2) in the CS group and
MT_24_: mean (geometric or arithmetic) of live ticks (categories 1 and 2) of the treated group.

Percent efficacy for the removal (48 h) count on each treatment group on each day was calculated as:

$$ Efficacy\ 48h\ \left(\%\right)=100\times \frac{\left({\mathrm{MC}}_{48}-{\mathrm{MT}}_{48}\right)}{{\mathrm{MC}}_{48}} $$

Where:

MC_48_: mean (geometric or arithmetic) of live ticks (categories 1–3) in the CS group and
MT_48_: mean (geometric or arithmetic) of live ticks (categories 1–3 for therapeutic efficacy) and live and dead ticks (categories 1–3 and 6 for residual efficacy) of the treated group.

The groups were compared using an ANOVA with a treatment effect after logarithmic transformation on the (tick count +1) data. Statistical significance was declared at a two-sided *p* value of 0.05.

### Guidelines

The study was conducted in accordance with the current and appropriate guidelines (CVMP 2000, 2007).

## Results

The BW and hair length of dogs were homogenous between groups (Table 2). The DPP and IP treatments delivered, respectively, 92 to 132 mg/kg BW and 83 to 112 mg/kg permethrin. The FM treatment delivered 8 to 12 mg/kg fipronil (Table 3).

**Table 2:** Dogs body weights and hair length

	Dogs body weight (kg)			Dogs hair length (mm)		
Group	Mean	Min	Max	Mean	min	Max
CS	13.7	10.8	18.4	23.4	12.0	61.3
DPP	12.7	10.8	15.6	24.8	12.5	59.5
FM	14.2	10.8	16.8	28.4	15.3	49.3
IP	13.3	11.2	15.0	19.3	12.8	24.5

**Table 3:** Concentrations of actives delivered individually in each group

Group	Actives	mg/kg BW*			mg/m^2^ BSA**		
		Mean	Min	Max	Mean	Min	Max
DPP	Dinotefuran	15.5	12.6	18.2	349	304	389
	Pyriproxyfen	1.4	1.1	1.6	31	27	35
	Permethrin	112.7	91.6	132.3	2,546	2,216	2,835
FM	Fipronil	9.4	8.0	12.4	221	198	266
	(S)-methoprene	8.5	7.2	11.2	199	178	239
IP	Imidacloprid	18.8	16.7	22.3	431	398	484
	Permethrin	93.8	83.3	111.6	2,154	1,990	2,421

*BW body weight, **BSA body surface area

The study was run in October and November which corresponds to the end of spring and beginning of summer in South Africa. The temperature inside the housing unit remained between 13.9 and 25.6 °C and the recorded relative humidity ranged from 17 to 74.7 %.

No adverse effects to treatment were observed in any of the treated dogs. The expected cosmetic effects, described as spiking with a wet paint brush effect, were noticed on all dogs 1 to 8 h after administration. Crystallization on the tips of hair was also observed in all groups: in 2 of the 8 dogs in the CS group and in 7 of the 8 dogs in the groups treated with DPP, IP, or FM. Twenty-four hours after administration, this cosmetic effect had disappeared in all groups.

### Therapeutic efficacy

In the CS group, all ticks found on dogs 3 and 4 days after infestation (1 and 2 days post-treatment) were live and attached. Three days after infestation, 17 to 32 live attached ticks were observed on each dog through palpation (Table 4). Four days after infestation, 20 to 30 live ticks were found on each dog and 10 of them were already showing conspicuous signs of engorgement (Table 5).

**Table 4:** *In situ* tick counts 24 h after weekly (days −2, 7, 14, 21, and 28) artificial infestations with adult *Rhipicephalus sanguineus* on mixed-bred dogs treated with a topical ectoparasiticide on day 0

Days	CS^A^		DPP		IP		FM	
	AM^B^	GM	AM	GM	AM	GM	AM	GM
1	24.1	23.7	21.8	20.9	22.8	19.7	21.9	21.2
8	26.4	25.1	0.1	0.1 ^1^	0.3	0.2 ^1^	2.0	1.5 ^1^ ^2^ ^3^
15	22.8	21.5	0.6	0.3 ^1^	1.5	0.7 ^1^	1.4	1.0 ^1^
22	27.1	25.9	0.9	0.5 ^1^	2.5	1.3 ^1^	5.8	4.7 ^1^ ^2^ ^3^
29	26.6	25.9	1.4	0.9 ^1^	4.5	2.2 ^1^	14.1	10.7 ^2^ ^3^

^A^ *CS* control solution, *DPP* dinotefuran, pyriproxyfen, and permethrin, *IP* imidacloprid and permethrin, *FM* fipronil and (S)-methoprene

^B^ *AM* arithmetic mean, *GM* geometric mean

^1^Group differed (*p* < 0.05) from the CS group

^2^FM group differed (*p* < 0.05) from DPP group

^3^FM group differed (*p* < 0.05) from IP group

**Table 5:** Tick removal counts 48 h after weekly (days −2, 7, 14, 21, and 28) artificial infestations with adult *Rhipicephalus sanguineus* on mixed-bred dogs treated with a topical ectoparasiticide on day 0

Days	CS^A^		DPP		IP		FM	
	AM^B^	GM	AM	GM	AM	GM	AM	GM
2	26.1	25.9	12.3	11.1 ^1^	14.3	11.9 ^1^	9.3	6.4 ^1^
9	26.8	25.1	0.1	0.1 ^1^	0.1	0.1 ^1^	0.4	0.3 ^1^
16	22.4	21.2	0.0	0.0 ^1^	1.4	0.4 ^1^	0.1	0.1 ^1^
23	27.9	26.6	0.1	0.1 ^1^	2.3	1.2 ^1^ ^2^	1.8	1.3 ^1^ ^3^
30	28.3	27.8	0.6	0.4 ^1^	4.3	2.0 ^1^	3.5	2.3 ^1^ ^3^

^A^ *CS* control solution, *DPP* dinotefuran, pyriproxyfen, and permethrin, *IP* imidacloprid and permethrin, *FM* fipronil and (S)-methoprene

^*B*^ *AM* arithmetic mean of tick numbers, *GM* geometric mean

^1^Group differed (*p* < 0.05) from the CS group

^2^IP differed (*p* < 0.05) from DPP

^3^FM differed (*p* < 0.05) from DPP

One day after administration, curative efficacy of the ectoparasiticides was below 20 % (Table 6). The number of live ticks found attached on dogs ranged from 14 to 31 in the DPP-treated group, from 3 to 34 in the IP-treated group and from 13 to 29 in the FM-treated group. Three live and free ticks were found on 2 dogs in the FM-treated group (Table 4).

**Table 6:** Efficacy at 24 h post-infestation and number of dogs free of live ticks after weekly (days −2, 7, 14, 21, and 28) artificial infestations with adult *Rhipicephalus sanguineus* on mixed-bred dogs treated with a topical ectoparasiticides on day 0

Days	DPP ^A^			IP			FM		
	AM ^B^	GM	TF	AM	GM	TF	AM	GM	TF
1	9.8	11.9	0/8	5.7	16.7	0/8	9.3	10.7	0/8
8	99.5	99.6	7/8	99.1	99.2	5/8	92.4	94.1	2/8
15	97.3	98.8	7/8	93.4	96.9	5/8	94.0	95.3	3/8
22	96.8	98.1	5/8	90.8	95.1	3/8	78.8	81.7	0/8
29	94.8	96.7	4/8	83.1	91.7	3/8	46.9	58.6	0/8

^A^ *DPP* dinotefuran, pyriproxyfen, and permethrin, *IP* imidacloprid and permethrin, *FM* fipronil and (S)-methoprene

^B^ *AM* efficacy calculated on arithmetic mean, *GM* efficacy calculated on geometric mean, *TF* number of dogs free of live-ticks

Two days after administration, efficacy of the ectoparasiticides ranged from 54.3 to 75.5 % (Table 7). The number of live attached ticks ranged from 6 to 24 ticks in the DPP-treated group, from 5 to 32 in the IP-treated group and from 1 to 20 in the FM-treated group. Up to 2 (IP), 3 (DPP), and 5 (FM) of these live ticks were found engorged per dog. In the treated groups, the GM tick counts were lower (*p* < 0.05) than in the CS group (Table 5). At both time points, it was not possible to detect any difference of GM tick counts between the products (Tables 4 and 5).

**Table 7:** Efficacy at 48 h post-infestation and number of dogs free of live ticks after weekly (days −2, 7, 14, 21, and 28) artificial infestations with adult *Rhipicephalus sanguineus* on mixed-bred dogs treated with a topical ectoparasiticides on day 0

Days	DPP^A^			IP			FM		
	AM^B^	GM	TF	AM	GM	TF	AM	GM	TF
2	53.1	57.1	0/8	45.5	54.3	0/8	64.6	75.5	0/8
9	99.5	99.6	7/8	99.5	99.6	7/8	98.6	99.0	6/8
16	100.0	100.0	8/8	93.9	98.3	7/8	99.4	99.6	7/8
23	99.6	99.7	7/8	91.9	95.4	3/8	93.7	95.0	2/8
30	97.8	98.5	5/8	85.0	92.8	3/8	87.6	91.8	2/8

^A^ *DPP* dinotefuran, pyriproxyfen, and permethrin, *IP* imidacloprid and permethrin, *FM* fipronil and (S)-methoprene

^B^ *AM* efficacy calculated on arithmetic mean, *GM* efficacy calculated on geometric mean, *TF* number of dogs free of live-ticks

Between 72 and 96 h after infestation (24 and 48 h post-treatment), the number of attached ticks decreased in the treated groups. In this time interval, whereas only one tick was missing from the CS group, 25 (DPP), 26 (IP), and 51 % (FM) of the ticks were not attached anymore in the permethrin and fipronil-treated groups, respectively.

### Residual efficacy

In the CS group, GM tick counts ranged from 21.5 to 25.9 as measured 24 h after infestation (Table 4) and from 21.2 to 27.8 as measured 48 h after infestation (Table 5). After palpation performed 24 h after infestation, all live ticks (from 12 to 41 per dog) were found attached, except one found free on day 8 and on day 29. None of the ticks were found engorged.

In the treated groups, residual efficacy evaluated by palpation 1 day after weekly infestation between day 8 and 29 was reduced from 99.6 to 96.7 % in the DPP-treated group, from 99.2 to 91.7 % in the IP-treated group, and from 94.1 to 58.6 % in the FM-treated group (Table 6).

The GM tick counts from day 8 to 29 were lower (*p* < 0.0001) in the DPP and IP groups as compared to the CS group (Table 4). In the FM group, tick counts that were statistically significantly (*p* < 0.05) lower than those of the CS group were detected only from day 8 to 22. On days 8, 22, and 29, the GM tick counts were lower (*p* < 0.01) in the DPP and IP-treated groups as compared to the FM-treated group. The number of dogs free of ticks decreased from 7 to 4 in the DPP-treated group, from 5 to 3 in the IP-treated group and from 3 to 0 in the FM-treated group (Table 6). In this last group, from day 22, none of the dogs were free of live ticks. Between day 8 and 29, free live and ranging ticks were only found in FM-treated group.

Two days after weekly infestation, residual efficacy, evaluated by removal of ticks between day 9 and day 30, ranged from 98.5 to 100.0 % in the DPP-treated group, from 92.8 to 99.6 % in the IP-treated group and from 91.8 to 99.6 % in the FM-treated group (Table 7). The GM tick counts from day 9 to 30 were lower (*p* < 0.0001) in the groups of dogs treated with DPP, IP, and FM as compared to CS group (Table 5). The GM tick counts in the DPP-treated group was lower (*p* < 0.05) than in the IP and FM-treated group on day 23 and lower (*p* < 0.05) than the FM-treated group on day 30. The number of dogs free of ticks decreased from 8 to 5 in the DPP-treated group, from 7 to 3 in the IP-treated group and from 7 to 2 in the FM-treated group (Table 7).

**Fig. 1:**
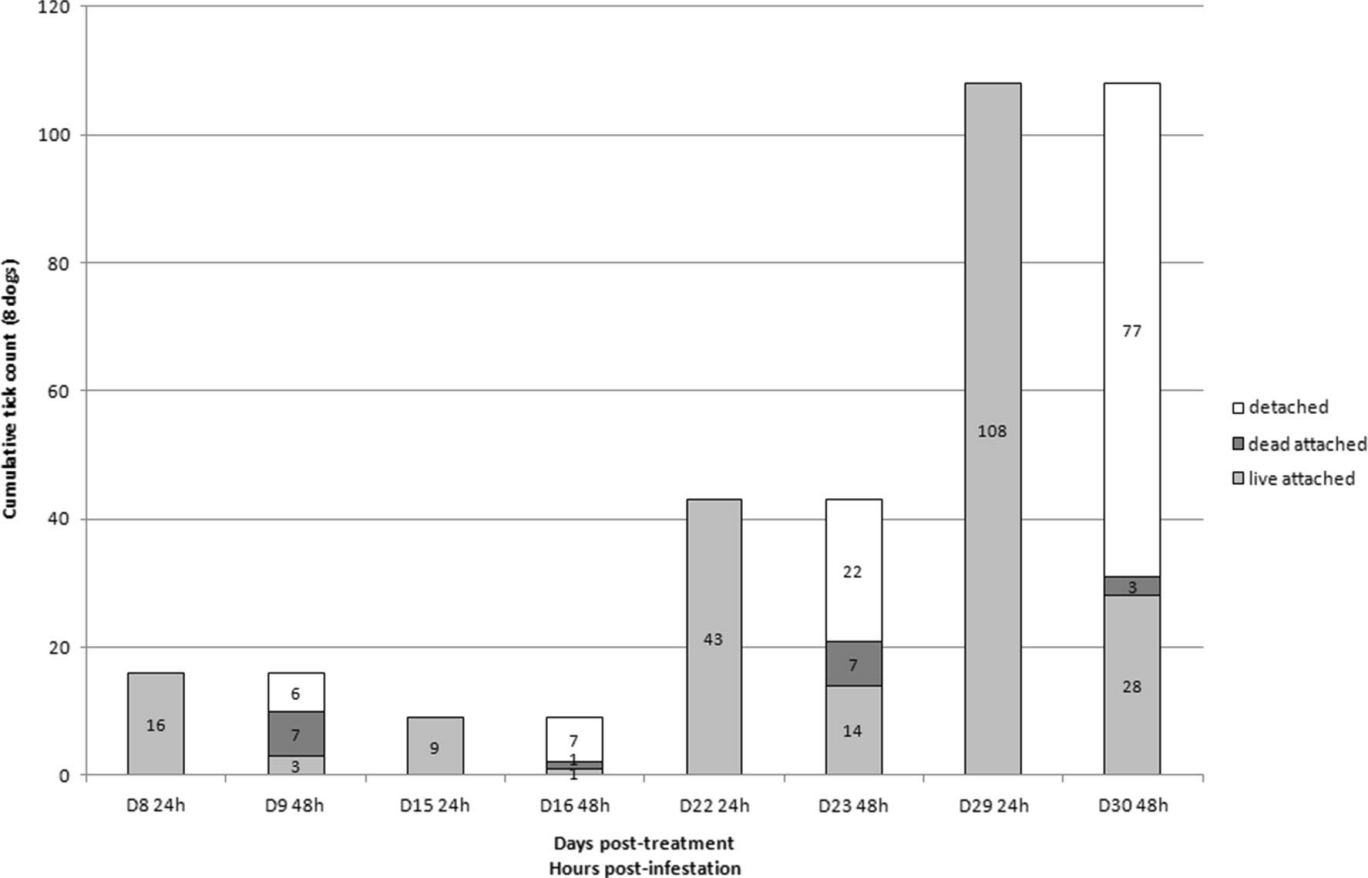
Evolution of the cumulative number of *Rhipicephalus sanguineus* ticks found live and attached 24 and 48 h, dead and attached 48 h, and detached between 24 and 48 h post-infestation in dogs (*n* = 8) treated with fipronil and (S)-methoprene formulation (Frontline plus) on day 0 and infested weekly with 50 ticks per individual

**Fig. 2:**
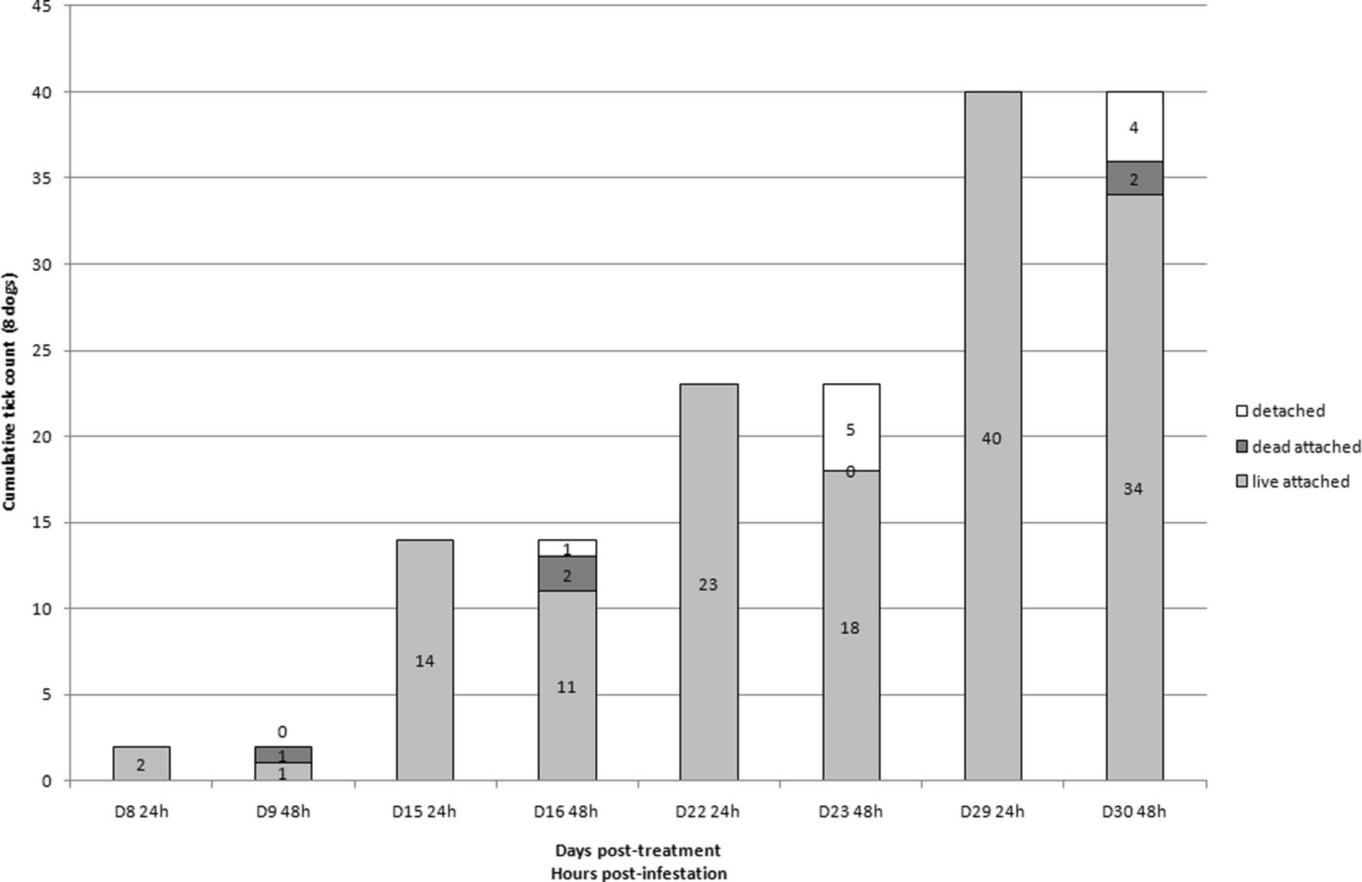
Evolution of the cumulative number of *Rhipicephalus sanguineus* ticks found live and attached 24 and 48 h, dead and attached 48 h and detached between 24 and 48 h post-infestation in dogs (*n* = 8) treated with imidacloprid and permethrin formulation (K9 Advantix) on day 0 and infested weekly with 50 ticks per individual

**Fig. 3:**
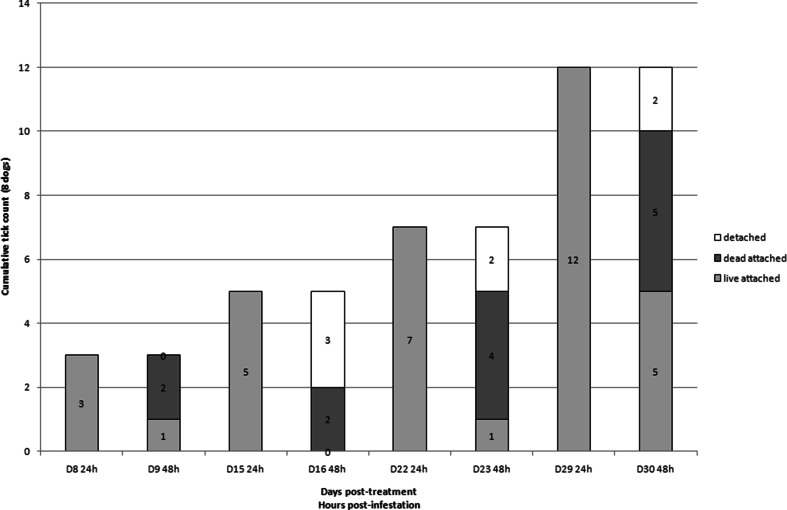
Evolution of the cumulative number of *Rhipicephalus sanguineus* ticks found live and attached 24 and 48 h, dead and attached 48 h, and detached between 24 and 48 h post-infestation in dogs (*n* = 8) treated with dinotefuran, pyriproxyfen, and permethrin formulation (Vectra 3D) on day 0 and infested weekly with 50 ticks per individual

**Fig. 4:**
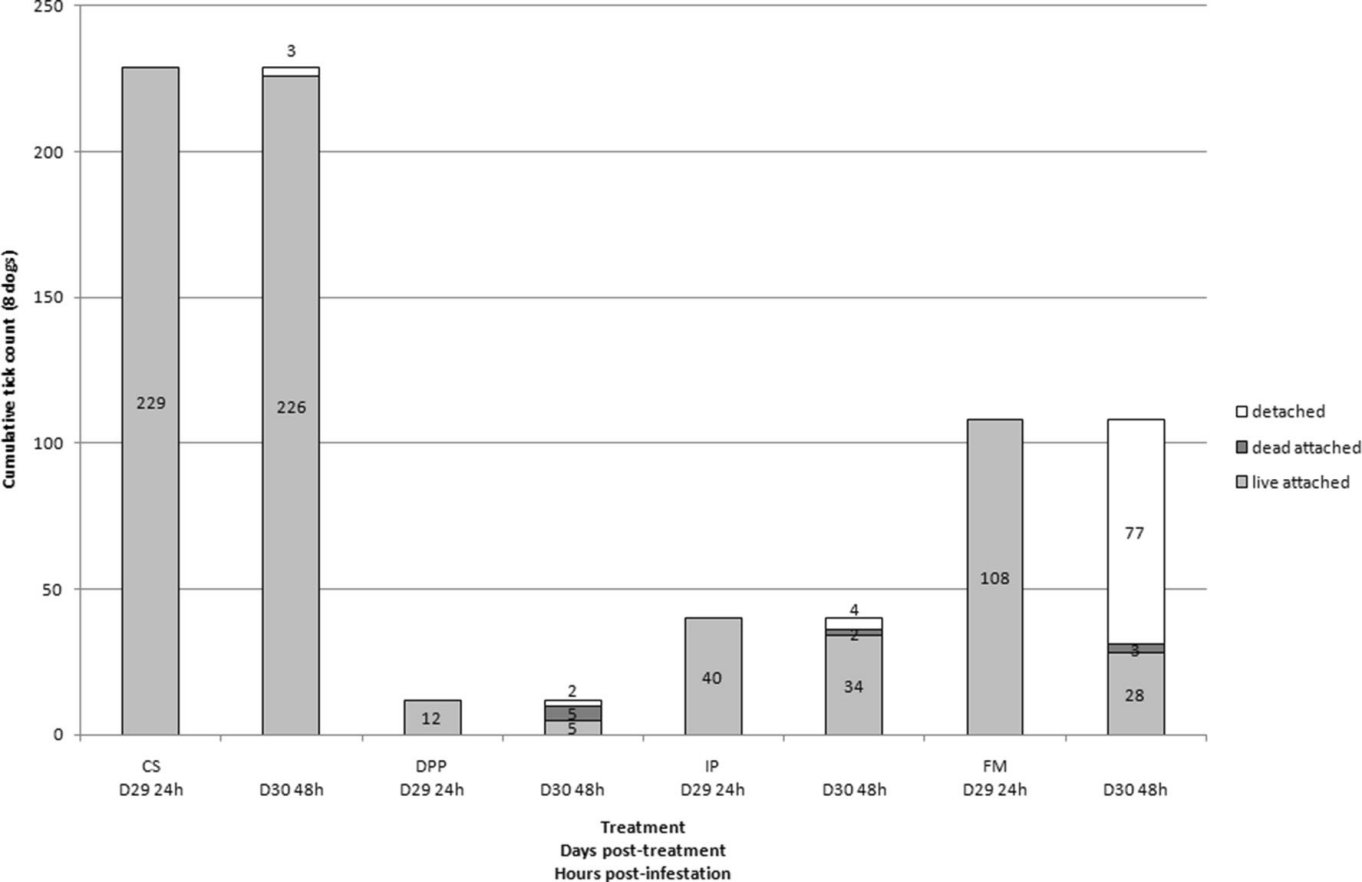
Number of *Rhipicephalus sanguineus* ticks found live and attached 24 and 48 h, dead and attached 48 h, and detached between 24 and 48 h after infestation with 50 ticks per dog (*n* = 8) on day 28 post treatment with CS, DPP, IP, or FM. *CS* control solution, *DPP* dinotefuran, pyriproxyfen, and permethrin formulation, *IP* imidacloprid and permethrin formulation, *FM* fipronil and (S)-methoprene formulation

All dogs treated with FM harbored live ticks 24 h after infestation on days 22 and 29 and most of those ticks were not attached anymore on dogs (51 and 71 % at days 22 and 29, respectively) on the second day post-infestation (Fig. 1). Some of the ticks were killed and found attached on the second day post-infestation (16 and 3 % at days 22 and 29, respectively). In dogs treated with IP, 78 to 85 % (from day 16 to 30) of the ticks were found live and attached at 24 h and were still live and attached 48 h post-infestation (Fig. 2). In dogs treated with DPP, there were fewer ticks on the dogs (12 vs. 108 in FM-treated group at day 29) and more parasites were killed (40 to 66 %) 24 and 48 h post-infestation (Fig. 3). Such differences were especially visible on day 29 (Fig. 4).

## Discussion

### Methodological considerations

The dogs were representative of mixed-breed animals and had summer coats of various lengths (Table 2). The range of hair length (12.0 to 61.3 mm) was in agreement with previous observations (8.5 to 66 mm) made on the guard hairs of mongrel dogs (Sato et al. 2006).

As demonstrated in the CS group, the tick challenges were successful during the whole duration of the study. The results obtained through the two methodologies, *in situ* measurement or final removal and combing, were very consistent. The maximal variation between *in situ* count and removal count was of 5 ticks.

Cosmetic effects were noticed in all the dogs after administration. It was first characterized by a wet effect on the hairs, mainly due to the deposit of the liquid as a spot-on. As this appearance disappeared between 8 and 24 h, we can state that the products dried within the 24 h post-administration. Crystallization on tips of the hairs was also observed 1 to 8 h after administration in most of dogs (7/8) in the treated groups and in 2 of the 8 dogs in the CS group. In the CS and the DPP group, crystallization of DPP’s vehicle is suspected even if frequency is lower in the CS group. Curiously, crystallization also occurred in FM, despite the presence of an anti-crystallization agent in the formula. No link could, however, be established between this cosmetic effect and the differences in efficacy between the products. We can consider that transient crystallization in hairs after application does not reduce the efficacy of the active ingredients.

### Therapeutic efficacy

In this study, the therapeutic efficacy, as measured 1 and 2 days after administration of the products, was evaluated 3 and 4 days post-infestation. For all products, efficacy levels were low and remained under the efficacy threshold of US and European authorities (i.e., 90 %). This was already observed on dogs already infested with *R. sanguineus* adult ticks on which treatment by IP exhibited a 74 % efficacy (Epe et al. 2003). These efficacy results present the acaricidal or detachment activity of the formulations on parasites that were already attached on the dogs. Indeed, when the products were applied, most ticks had enough time to find their preferred location on the dogs and arrest themselves for attachment and initiation of the feeding process. In rabbits, which are not the usual host for this parasite, about fifteen percent of adult *R. sanguineus* ticks infested directly on ears were shown to attach within the first hour post-infestation. This proportion increased almost linearly, reaching 100 % in 48 h (Socolovschi et al. 2009). In our study, dogs were infested with ticks 48 h prior to treatment. We can therefore consider that most ticks were attached before being exposed to the actives. This is a challenging situation as the level of exposure of the ticks to the actives is reduced. Moreover, spot-on products are applied on the back of the dogs and the actives need time to spread on the body surface and coat to reach the most distal parts of the dogs. Despite this, whereas 10 ticks were already showing signs of obvious engorgement in the CS group, only 5 ticks were in this stage in the groups treated with the permethrin-based formulations. In the FM-treated group, a total of 7 ticks were found showing signs of obvious engorgement. Moreover, from 25 (permethrin) to 51 % (fipronil) of the attached ticks counted 72 h after infestation (24 h post-treatment) were not found anymore from the dogs and had possibly detached themselves as observed at the 96 h count (48 h post-treatment).

The dogs in our experiment were administered a minimum dose of 94 (IP) to 113 mg/kg BW (DPP) permethrin. When administered about twice this dose, at 50 mg/kg BW of permethrin, the concentration in hairs collected from the legs and back of dogs ranged between 132 to 3,793 μg of permethrin per gram of hairs (Lüssenhop et al. 2011a). The LC50 of permethrin for *R. sanguineus* semi-engorged females was established at 1,549–2,675 ppm after 5 min exposure by immersion of adults (Roma et al. 2009). However, very low doses of both permethrin (5 min immersion in 206–2,062 ppm Nodari et al. 2011, 2012a, b) and fipronil (2–3 min in 1–10 ppm Pereira et al. 2009, 2011) were shown to accelerate the degenerative process of salivary glands, even leading to an inhibition of saliva secretion at larger doses. We can hypothesize that the doses of permethrin in our study may not be sufficient to kill 90 % of the ticks but that these doses provided a substantial protection against blood feeding process. To reduce the risk of disease transmission, the search and removal of attached ticks should have always been advisable before administration of an acaricidal product in dogs.

### Residual efficacy

Residual efficacy was assessed weekly in situations where the parasites must enter the coat of the dogs and move on the animal’s skin up to their location for feeding, increasing both their level of metabolism and their exposure to already treated skin and hairs.

At several time-points (days 8, 22, 23, 29, and 30), the permethrin-based formulations exhibited a higher residual efficacy than the fipronil-based formulation. Even if permethrin and fipronil are both recognized for their neurotoxicity on arthropods, these actives have different modes of action. Permethrin is a synthetic non-α-cyano-pyrethroid while fipronil is a phenylpyrazole. As demonstrated on insects, the actives have different targets: fipronil blocks glutamate-activated chloride channels whereas permethrin acts on sodium channels. The onset of activity of both actives is also known to differ widely. Whereas permethrin was shown to be the fastest acaricide, fipronil was identified as the slowest one (White et al. 2004). Indeed, *in vitro*, fipronil requires, from 18 to 24 h, to exhibit an inhibition of motility of stimulated *R. sanguineus* ticks. These results were obtained with doses of fipronil ranging from 0.325 to 1.300 μg/cm^2^ of surface (Prullage et al. 2011). From 21 days after treatment of 12 kg BW Beagles with a topical dose of 10 mg/kg BW of fipronil, the amount of fipronil detected was <0.100 μg/cm^2^ of body surface area in the treated and lumbar zones of dogs. At 29 days post-treatment, it was only 0.05 μg/cm^2^ in the lumbar zone of dogs (Cochet et al. 1997). In our study, the average BW of dogs in the FM group was 14.2 kg and the average fipronil dose was 9.4 mg/kg BW. Concentration of fipronil available on the skin and hairs of these dogs was hence far below the required dose for efficacy on ticks within 24 h of exposure (Prullage et al. 2011). This probably explains why the efficacy of FM dropped so dramatically from days 22 to 29 and why so many ticks were able to attach to dogs within 24 h after reinfestation. Finally fipronil and permethrin exhibit considerable differences regarding repellency and knock-down effect. Fipronil exhibited a noticeable detachment effect on the ticks between 24 and 48 h after infestation (day 29, 77/108). Only a small proportion of ticks were found dead and still attached on the dogs (day 29, 3/108). As these released ticks were not monitored, their viability was not assessed. Contrary to fipronil, permethrin is not only acaricidal but also exhibits repellency and knock-down effects. These properties are key factors for the prevention of tick attachment on the host and contributed clearly to the efficacy of the permethrin-based combination in this study. Field trials comparing IP and FM already demonstrated that permethrin-based combinations prevent tick attachment through repellency (Young et al. 2003; Dryden et al. 2006). Tick repellency was observed *in vitro* and on dogs under controlled conditions as the result of avoidance behavior and hot-foot reactions induced by permethrin (Mehlhorn et al. 2003). It was also demonstrated that the repelled *R. sanguineus* ticks, falling from the treated dogs within the first 10 min of exposure, were killed by permethrin in a few hours (Dryden et al. 2006). The results of our study confirmed that permethrin-based formulations provide a high level of prevention against tick attachment for 1 month.

A much lower number of ticks were found on dogs in the DPP group (12) than in the IP group (40) after the last infestation (Fig. 1). On day 23, GM tick count was statistically lower in the DPP-treated group than in the IP-treated group. Differences in formulation were already shown to influence efficacy levels of permethrin-based combinations against ticks on dogs, as expressed as a number of total attached ticks (Lüssenhop et al. 2011b). The lower dose of permethrin applied to the IP-treated dogs and the differences in formulation between the two combinations may have contributed to reduce the efficacy of this combination.

## Conclusions

This experiment, conducted on mixed-bred dogs of various hair lengths, demonstrates that DPP and IP combinations provide a better monthly lasting protection against *R. sanguineus* infestations than FM. Whereas both DPP and IP are permethrin-based actives, only DPP exhibited satisfactory protection against ticks at every time-point for one month. The DPP formulation, which produced a high (>96 %) residual efficacy, should be considered as a reliable veterinary prevention tool against infestations by the brown dog tick *R. sanguineus*.
